# Purification of antibody fragments via interaction with detergent micellar aggregates

**DOI:** 10.1038/s41598-021-90966-1

**Published:** 2021-06-03

**Authors:** Gunasekaran Dhandapani, Ellen Wachtel, Ishita Das, Mordechai Sheves, Guy Patchornik

**Affiliations:** 1 Department of Chemical Sciences, Ariel University, 70400 Ariel, Israel; 2 Faculty of Chemistry, Weizmann Institute of Science, 76100 Rehovot, Israel

**Keywords:** Isolation, separation and purification, Protein purification

## Abstract

The research described in this report seeks to present proof-of-concept for a novel and robust platform for purification of antibody fragments and to define and optimize the controlling parameters. Purification of antigen-binding F(ab′)_2_ fragments is achieved in the absence of chromatographic media or specific ligands, rather by using clusters of non-ionic detergent (e.g. Tween-60, Brij-O20) micelles chelated via Fe^2+^ ions and the hydrophobic chelator, bathophenanthroline (batho). These aggregates, quantitatively capture the F(ab′)_2_ fragment in the absence or presence of *E. coli* lysate and allow extraction of only the F(ab′)_2_ domain at pH 3.8 without concomitant aggregate dissolution or coextraction of bacterial impurities. Process yields range from 70 to 87% by densitometry. Recovered F(ab′)_2_ fragments are monomeric (by dynamic light scattering), preserve their secondary structure (by circular dichroism) and are as pure as those obtained via Protein A chromatography (from a mixture of F(ab′)_2_ and Fc fragments). The effect of process parameters on Ab binding and Ab extraction (e.g. temperature, pH, ionic strength, incubation time, composition of extraction buffer) are reported, using a monoclonal antibody (mAb) and polyclonal human IgG’s as test samples.

## Introduction

The importance of monoclonal antibodies (mAbs) as therapeutic agents is steadily increasing and is reflected by the fact that in 2015 alone, more than 1000 mAbs were evaluated for their clinical use^1^; approx. 300 new mAbs are being assessed each year for their effectivity^1^. In parallel with the clear impact of mAbs on medicine, much interest has also been expressed in the use of fragments of antibodies (Ab-fragments), which generally lack the crystallizable fragment (Fc) domain^2–4^ and as such are significantly lower in molecular weight with respect to intact mAbs. Examples of Ab-fragments include: the antigen-binding fragments—Fab & F(ab′)_2_; the single-chain variable fragment (scFv); the single variable heavy-chain domain (V_H_) and the single variable light-chain domain (V_L_)^1^. These Ab-fragments have physiochemical properties that differ from intact Abs, which translate into (i) a larger number of binding events per mass of protein; (ii) an improved tissue penetration capability; (iii) less viscous formulations that render administration less painful; (iv) shorter half-life in serum; and (v) less likelihood of triggering an immune response^3^. As a result, Ab-fragments have been coupled with (a) dyes—for diagnostic applications^3,5^; (b) enzymes, radioisotopes and toxins—for cancer therapy^6–10^; and (c) vesicles and nano-particles—for enhancing drug delivery^11–13^.

Unlike the use of Protein A as a general, highly efficient and robust ligand for antibody purification^14^, no analogous general ligand exists for Ab-fragments, primarily due to the absence of the Fc domain to which Protein A binds with high affinity^15^ and specificity^16^. Thus, the absence of the Fc domain in most Ab-fragments is closely correlated with the difficulty in purifying this diverse class of proteins^17^. Pharmaceutical companies have consequently been working to identify a universal ligand capable of efficiently binding most types of Ab-fragments. A potentially general ligand was suggested to be Protein L^1^. This bacterial protein (76–106 KDa) is found in *Peptostreptococcus magnus*^18^ and contains 4–5 highly homologous extracellular binding sites that recognize the kappa light chain of most repesentatives of antibody subclasses^19^. Therefore, Protein L was immobilized on chromatographic resins and was shown to allow purification of diverse Ab-fragments, including Fab^20,21^, scFv^22–24^ and a single antibody domain^25,26^. However, the costs associated with the use of Protein L affinity chromatography for industrial-scale purification of therapeutic grade Ab-fragments, are high and more economic alternatives are required^1^.

We have recently presented a novel purification platform for intact immunoglobulin G (IgG) that does not rely on chromatographic media or ligand^27–29^. This approach makes use of non-ionic detergent (e.g. Tween-20, Brij C-10, Triton X-100) micelles conjugated via the [(bathophenanthroline)_3_:Fe^2+^] amphiphilic complex. Such detergent aggregates were shown to quantitatively capture diverse IgG’s from human, mouse, rabbit, or sheep sera, reject hydrophilic proteins and allow extraction of captured IgG’s from the aggregates without concomitant impurity co-extraction or aggregate dissolution^27–29^.

**Figure 1:**
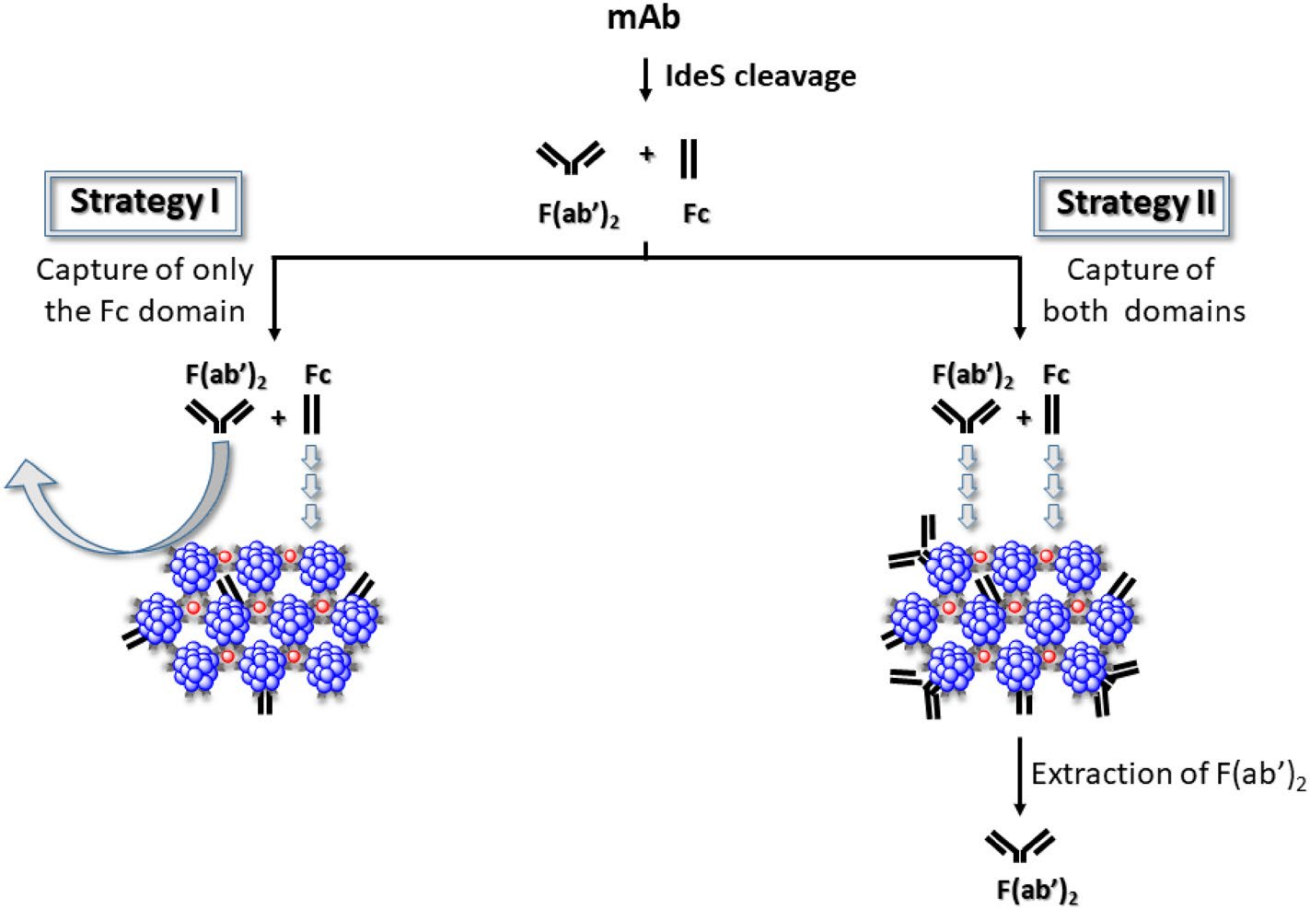
Illustration of two alternative purification strategies of antibody fragments. Two approaches for separation of F(ab′)_2_ fragments from Fc fragments were evaluated. In Strategy I—nonionic detergent aggregates bind almost quantitatively the Fc domain and leave a fraction of the F(ab′)_2_ segments in the supernatant. In Strategy II—detergent aggregates bind almost quantitatively both domains but allow extraction of primarily the F(ab′)_2_ segment at pH 3.8, leaving the majority of the Fc fragments bound to the detergent aggregates.

The observed quantitative binding of diverse IgG's to detergent aggregates raised the possibility of the potential utility of this platform for isolation/purification of antibody fragments. As a first step towards such a goal, a monoclonal antibody (mAb), subjected to enzymatic cleavage by IdeS, was chosen as most suitable for proof-of-concept. IdeS is known to cleave IgG’s to the crystallizable (Fc) fragment domain and the antigen binding fragment (F(ab′)_2_)^30–32^. Since hydrophobicity appears to strengthen detergent/IgG interaction, if either the Fc or the F(ab′)_2_ fragment is relatively more hydrophobic, this should in principle permit their separation from one another. If the more hydrophobic domain is located in the Fc fragment, then we may foresee two distinct purification strategies. (1) The Fc fragment is captured by the detergent aggregates, while the pure F(ab′)_2_ domain remains in the supernatant (Fig. 1, Strategy I). (2) Both fragments are captured and the F(ab′)_2_ domain is extracted from the aggregates leaving the Fc segment bound to the detergent matrix\scaffold (Fig. 1, Strategy II). The experimental results presented below provide evidence for platform efficiency for purifying F(ab′)_2_ fragments, also in the presence of *E. coli* lysate; it has not been tested with other antibody fragments (e.g. Fv, scFv) commonly employed in clinical settings.

### Experimental

#### Materials

Glycine, valine, isoleucine, leucine, arginine, lysine, histidine, glutamic acid, iron(II) chloride, sodium chloride, sodium dodecyl sulfate, polysorbate 20, polysorbate 40, polysorbate 60, polysorbate 80, Brij C-10, Brij O-20, Brij S-100, Triton X-100, Protein A HP Spin-Trap, human IgG, rabbit IgG, Ex-CELL 610-HSF medium were all purchased from Sigma-Aldrich (St. Louis, MO). Bathophenanthroline was from GFS Chemicals. The mAb—cB72.3 Sarto QFT was kindly provided by Lonza, Switzerland; however, we were not provided with the amino acid sequence, the identity of the target antigen, nor the expression cell culture. Antibody digestion was performed using the FragIT (Genovis) kit containing the IdeS cysteine protease.

## Methods

### Strategy I

#### IdeS digestion

Antibody digestion was performed using the FragIT (Genovis) kit in a total volume of 100 µL containing: 20 mM NaCl, 10 NaPi (pH 7.4) and 0.5 mg (total amount) of the target mAb (CB72.3 Sarto QFT). After 3 h of incubation at 37 °C, centrifugation (15,800 × *g*, 2 min, Eppendorf Microfuge 5424-R) was applied and the sample was either used immediately or stored at −20 °C.

#### Preparation of Brij S-100 detergent aggregates

Detergent aggregates were obtained by mixing equal volumes of medium A and B as follows: **medium A** was prepared by the addition of 30 μL of the hydrophobic chelator bathophenanthroline (20 mM in methanol) to 270 μL of 0.05 mM Brij S-100 in DDW with vigorous vortexing. An equal volume of **medium B**, containing 1 mM FeCl_2_ in 20 mM NaCl was then added to medium A with vigorous vortexing. After 5 min of incubation at room temperature 23 μL of 5 M NaCl were added and the system was further incubated for an additional 5 min at 4 °C. A short spin followed (15,800 × *g*, 5 min, Eppendorf Microfuge 5424-R), the supernatant discarded and the aggregates were washed with 100 μL of DDW. Repetition of the spin resulted in red Brij S-100 aggregates that were used for purification of antibody fragments.

#### Purification protocol

mAb (67 µL) digested with IdeS were mixed with hybridoma serum-free media (33 µL) and incubated for 10 min at room temperature with freshly prepared Brij S-100 aggregates (100 µL). Centrifugation was applied (15,800 x *g*, 5 min) and the supernatant was analyzed by SDS-PAGE.

### Strategy II

#### IdeS digestion

The antibody cleavage protocol was identical to the one described above for strategy I, except for the use of 150 mM NaCl during cleavage.

#### Preparation of Tween-60 detergent aggregates

Detergent aggregates were obtained according to the same protocol used with Brij S-100 aggregates except for the use of 270 μL 0.062 mM Tween-60 in DDW instead of 270 μL of 0.05 mM Brij S-100.

#### Purification protocol

mAb (67 µL), digested with IdeS, and 4% PEG-6000 were mixed with hybridoma serum-free media (33 µL) and incubated with Tween-60 aggregates for 10 min at room temperature. Centrifugation was applied (13 K, 5 min) and the supernatant discarded. The pellet was resuspended in cold 20 mM NaCl (100 µL) and centrifugation was repeated (15,800 × *g*, 5 min, Eppendorf Microfuge 5424-R). Captured F(ab′)_2_ fragments were extracted from the pellet by incubating the latter in 50 mM Val, 150 mM NaCl (pH 3.8) for 10 min at room temperature. A short spin followed repeated (15,800 × *g*, 5 min, Eppendorf Microfuge 5424-R), the supernatant containing the extracted F(ab′)_2_ fragments was collected and analyzed in SDS-PAGE.

### Circular dichroism (CD) spectroscopy

Purified F(ab′)_2_ fragments were subjected to CD analysis using a Chirascan CD spectrometer (Applied Photophysics). CD spectra report ellipticity (θ), proportional to the difference in absorbance of left and right circularly polarized light [θ = 3300° (A_L_ − A_R_)] as a function of wavelength. A quartz cell of path length 0.1 cm was used for the measurements. The CD spectra were recorded with 2 nm bandwidth resolution in 1 nm steps at 25 °C. The collected CD spectra were corrected for baseline distortion by subtracting a reference spectrum of the corresponding buffer solution.

### Densitometry

Bands present in Coomassie stained gels were quantified using the EZQuant program. http://www.ezquant.com/en/.

### Dynamic light scattering (DLS)

Purified F(ab′)^2^ fragments (0.5–1.0 mg/mL) in 50 mM glycine and 30 mM NaCl at pH 3.8, were neutralized to pH 7 with 1.5 M Tris mM, pH 7.5, and centrifuged at 15,800 × *g*, Eppendorf Microfuge 5424-R for 20 min. The size distributions of the samples were determined using the auto correlation spectroscopy protocol of the Nanophox instrument (Sympatec-GmbH, Germany).

## Results and discussion

**Figure 2:**
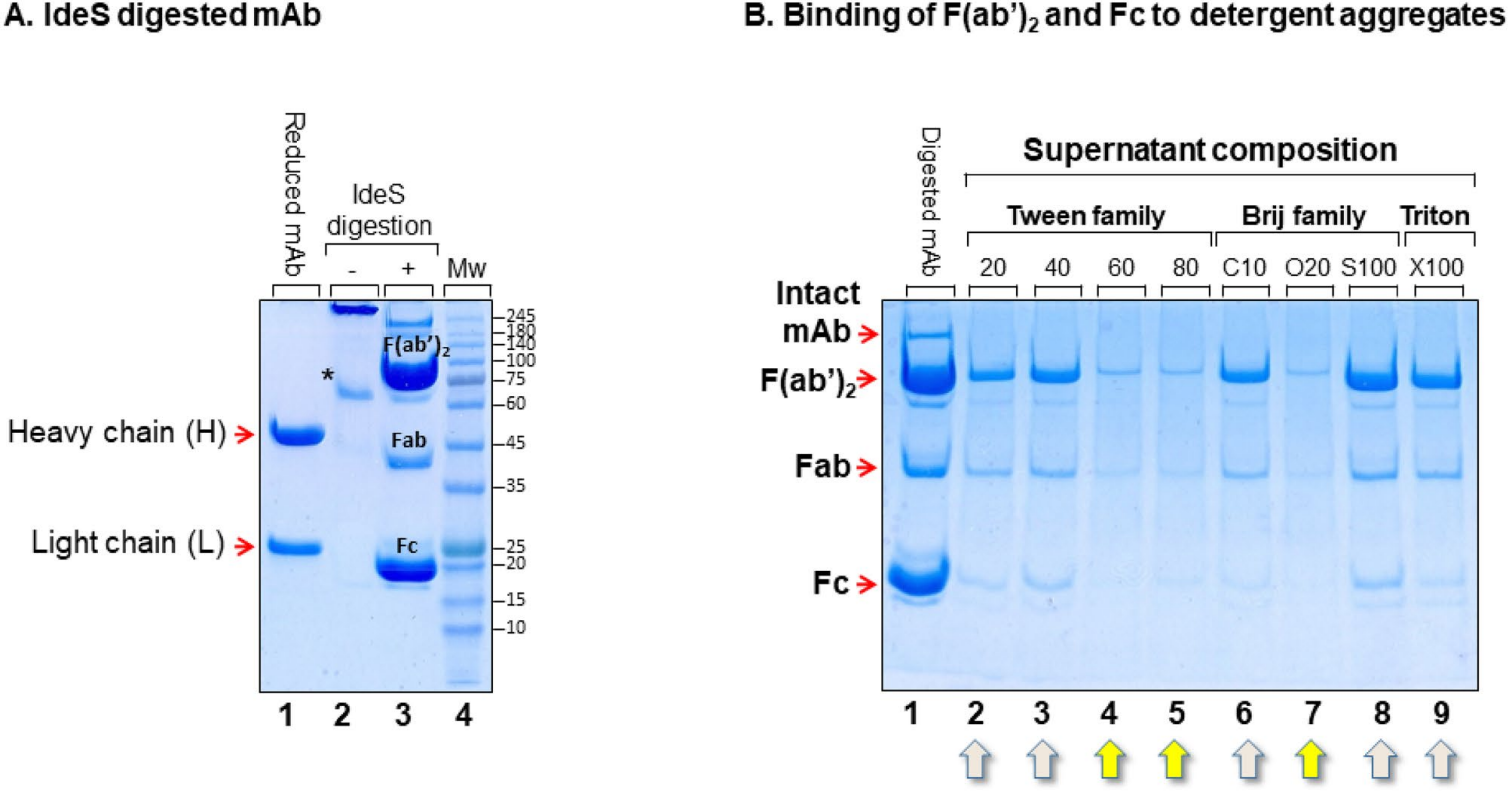
(**A**) Digestion of mAb with papain. Lane 1: reduced mAb; lane 2: unreduced mAb; lane 3: IdeS digested mAb. The asterisk indicates a minor antibody fragment with unknown identity that seems to contain disulfide bonds due to its disappearance upon reduction in lane 1. (**B**) Supernatant composition after brief incubation of detergent aggregates (as indicated) with IdeS cleaved mAb. Yellow arrows point to detergent aggregates that bind all three major fragments (i.e. Fab, F(ab′)_2_ and Fc). Grey arrows point to detergent aggregates that preferentially bind the Fc domain. Gels are Coomassie stained.

Digestion of mAb cB72.3 with IdeS produced two major fragments at ~ 75–100 KDa and ~ 20 KDa , representing the F(ab′)_2_ and Fc fragments, respectively (Fig. 2A—lane 3); a minor band at ~ 40–42 KDa representing the Fab fragment (derived presumably from reduction of F(ab′)_2_ by 2-mercaptoethanol present during SDS-PAGE analysis); and an additional minor band at MW > 150 KDa that may represent the intact mAb or an aggregate. This Ab-fragment mixture served for the study of Strategies I and II (Fig. 1). When the Ab-fragment mixture was incubated for 10 min with nonionic detergent aggregates, containing the [(bathophenanthroline)_3_:Fe^2+^] amphiphilic complex, only Tween-60, Tween-80 or Brij-O20 detergents were observed to efficiently bind the Fc and F(ab′)_2_ fragments (Fig. 2B—lanes 4,5 and 7, respectively). Aggregates composed of Tween-20, Tween-40, Brij-C10, Brij-S100 and Triton X-100 were found to trap the Fc domain (Fig. 2B—lanes 2,3,6,8 and 9, respectively). Since within the 4 members of the Tween family (or within the 3 members of the Brij family), no clear trend with respect to Ab-fragment binding preference was observed (Fig. 2B), detergents that appeared to suit either Strategy I or II were investigated further.

**Figure 3:**
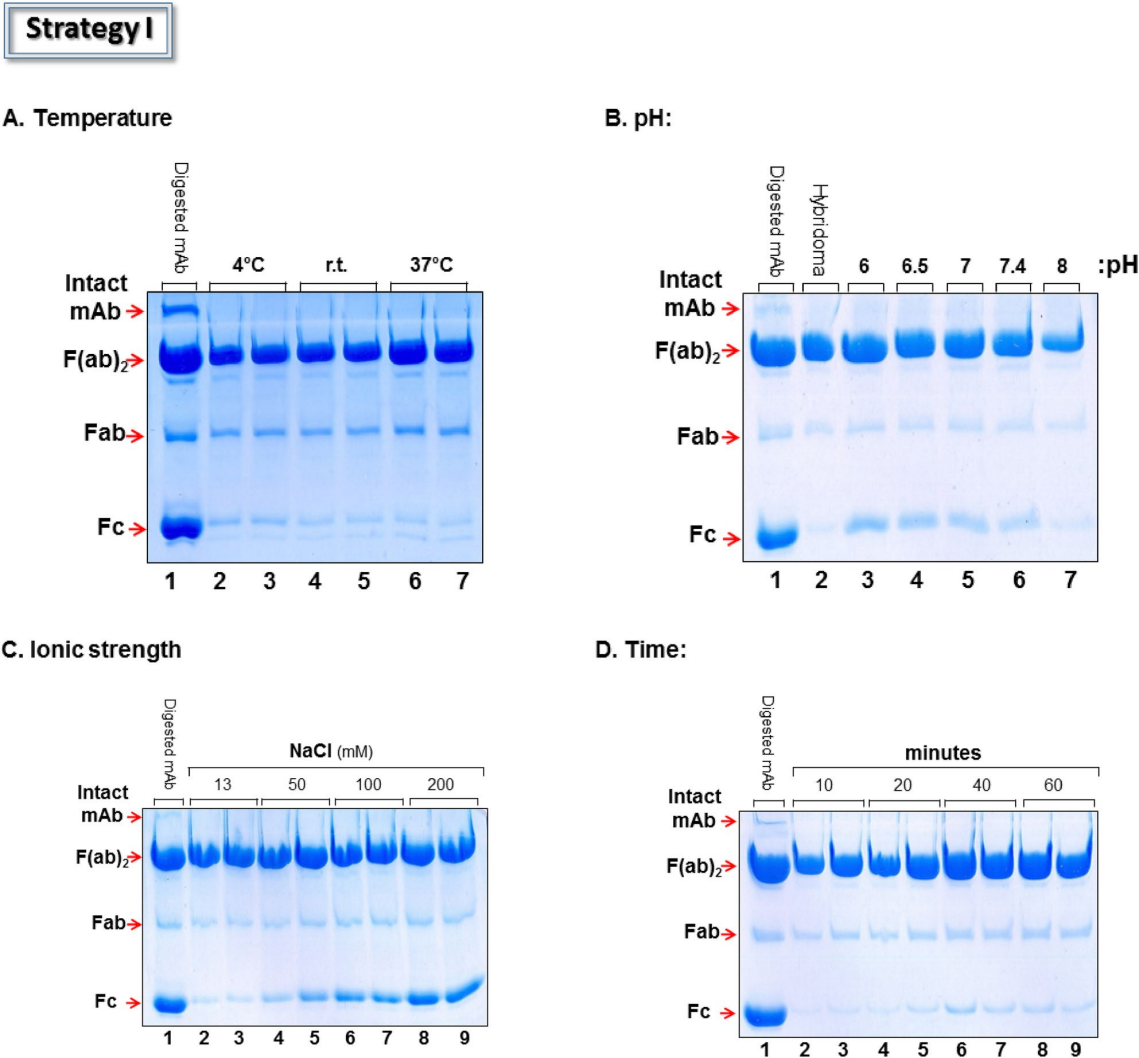
Parameters affecting strategy I. (**A**) Effect of temperature on the binding of Ab-fragments to Brij S-100 aggregates. Samples represent the supernatant composition after brief incubation (10 min.) with the detergent aggregates. (**B**) As in A, but at pH values indicated. (**C**) As in A, but at salt concentrations indicated. (**D**) As in A, but at pH 7.4, 13 mM NaCl and indicated time points. Gels are Coomassie stained. *E. coli* lysate was not added.

Accordingly, Strategy I was studied with Brij-S100 under a variety of working conditions (Fig. 3). Incubation at 37 °C led to the largest number of Fab and F(ab′)_2_ fragments in the supernatant along with a relatively low concentration of the Fc domain (Fig. 3A, lanes 6–7), while between 4 and 25 °C, the intensity of the F(ab′)_2_ band decreased, indicating stronger binding of this fragment to Brij-S100 aggregates (Fig. 3A, lanes 2–5). This finding may reflect the weakening effect of temperature on non-covalent interactions between the F(ab′)_2_ domain and the detergent aggregates. The higher the temperature, the weaker the interactions and hence, more of the F(ab′)_2_ fragment in the supernatant. Despite the relatively strong presence of the F(ab′)_2_ fragment in the supernatant (71–80%, by densitometry), small amounts of the Fc domain were observed as well (< 3%, by densitometry) (Fig. 3A, lanes 6–7).

In an attempt to further improve purity and yield, we investigated the impact of pH. Values around neutrality seemed to be good (Fig. 3B, lanes 4–6) whereas at pH 8, process suppression was clearly observed (Fig. 3B, lane 7). The contribution of ionic strength to process efficiency showed a clear trend (Fig. 3C). Low ionic strength (e.g. 13 mM NaCl at pH 7.4) led to minimal amounts of the Fc fragment in the supernatant whereas at higher salt concentration (e.g. 200 mM NaCl at pH 7.4) the amount of Fc increased dramatically (Fig. 3C, lanes 8–9). We hypothesize that the greater amount of Fc in the supernatant at high NaCl concentration derives, at least in part, from the known general trend of salts to increase the water solubility of proteins^33^. Once the optimal pH (pH 7.4) and salt concentration (13 mM) were defined, the optimal incubation time was sought and found to be ~ 40 min (Fig. 3D, lanes 6–7) leading to recovery yields for (F ab′)_2_ in the range of 84–90% (by densitometry). Though process optimization led to good recovery yields, trace amounts of the Fc fragment were still evident (Fig. 3D, lanes 6–9) and additional efforts (e.g. changing metal, detergent) failed to improve results (not shown).

**Figure 4:**
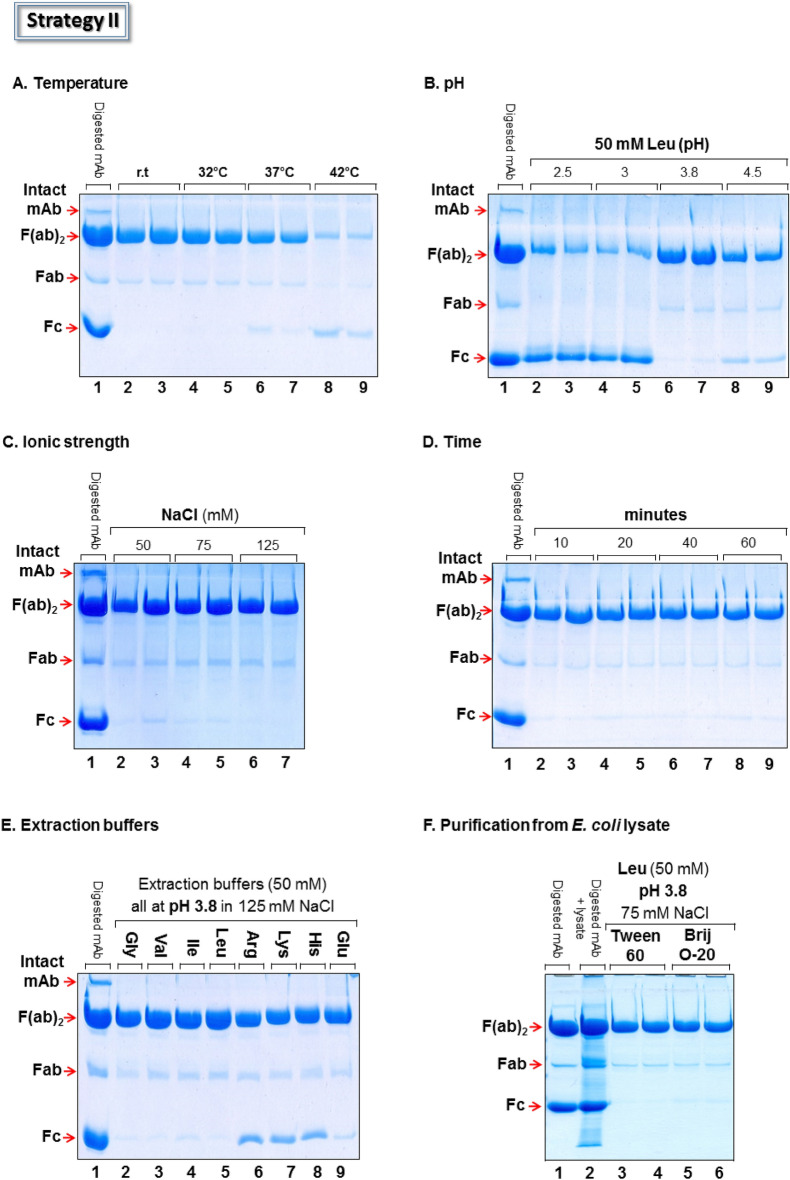
Parameters affecting Strategy II. (**A**) Effect of temperature on the extraction efficiency of Ab-fragments from Tween-60 aggregates at pH 3.8 in the presence of 50 mM leucine and 125 mM NaCl. (**B**) As in A, but at indicated pH values. (**C**) As in A, but at indicated salt concentrations. (**D**) As in A, but at pH 7.4, 13 mM NaCl and indicated time points. **E** As in A, but with indicated buffers and salt after 10 min at room temperature. (**F**) Effect of *E. coli* lysate on process efficiency. Lane 1: IdeS digested mAb; lane 2: mixture of lane 1 and *E. coli* lysate representing the protein preparation added to the conjugated detergent aggregates; lanes 3–6: F(ab′)_2_ recovered from Tween-60 or Brij-O20 aggregates, respectively and under the extraction conditions indicated. Gels are Coomassie stained.

Encouraged by the above, we focused on assessing the purification strategy II (Fig. 1, Strategy II) using Tween-60 aggregates; these proved to be efficient in capturing both the Fc and F(ab′)_2_ fragments (Fig. 2, lane 4). The challenge was to define conditions that would allow extraction of captured F(ab′)_2_ fragments from Tween-60 aggregates without parallel co-extraction of the Fc domain or aggregate dissolution. Extraction experiments were initially carried out at different temperatures in the presence of 50 mM Leu (pH 3.8) and 125 mM NaCl; a clear trend was observed (Fig. 4A). Temperatures near ambient and up to 32 °C led to the highest extraction efficiency (Fig. 4A, lanes 2–5) whereas at higher temperature (37–42 °C), extraction yields progressively decreased (Fig. 4A, lanes 6–9). Moreover, extraction at temperatures between 25 and 32 °C recovered F(ab′)_2_ fragments that were not contaminated by observable amounts of the Fc fragment (Fig. 4A, lanes 2–5). The greater purity of recovered F(ab′)_2_ fragments represented an advantage of Strategy II over Strategy I. It is unclear to us why extraction efficiency at 42 °C is inferior to lower temperatures.

Once the optimal temperature was defined as 25 °C, we aimed at improving the extraction yield of the F(ab′)_2_ fragment. We studied the effect of pH using the amino acid leucine (50 mM) as the extracting buffer (Fig. 4B). The most effective pH was found to be pH 3.8 while lower pH values (pH 2.5–3) led to a dramatic increase in the extraction of the Fc domain (Fig. 4B, lanes 2–5). At higher pH values (e.g. pH 4.5), extraction was suppressed (Fig. 4B, lanes 8–9). These changes may derive from the effect of pH on the water-solubility of the Fc and F(ab′)_2_ domains, which in turn, weakens or strengthens the interaction of these domains with the detergent matrix.

Ionic strength demonstrated again its role in affecting the binding of the Fc to the detergent aggregates. Moderate NaCl concentration (125 mM) was needed to suppress co-extraction of the Fc fragment (Fig. 4C, lanes 6–7) whereas at lower salt concentrations (50–75 mM), small amounts of co-extracted Fc was observed (Fig. 4C, lanes 2–5). Thus, the working pH value, combined with the identity of the detergent used, seem to determine optimal salt conditions needed to suppress coextraction of the Fc segment. For example, neutral pH and 13 mM NaCl were needed to suppress parallel extraction of the Fc fragment from Brij S-100 aggregates (Fig. 3C, lanes 2–3), whereas for Tween-60 aggregates at pH 3.8, 125 mM of NaCl were required (Fig. 4C, lanes 6–7). With respect to extraction time, 10 min were found to be optimal (Fig. 4D, lanes 2–3) and represented an additional advantage of Strategy II over Strategy I (requiring ~ 40 min, Fig. 3D, lanes 6–7).

The finding that ionic strength weakened the binding affinity of the Fc domain during the capturing step in Strategy I (Fig. 3C) but had no effect on the Fc segment during the extraction step in Strategy II (Fig. 4C) was unexpected; if salt lowers the binding affinity between the Fc domain and the aggregates during the capturing step, then salt should also promote the extraction of the Fc domain during the extraction step. The fact that this was not observed implies that the lower pH value (pH 3.8) during the extraction step (rather than ~ pH 7 in the binding step) is able to overcome the impact of salt molarity, strengthens the binding affinity of the Fc segment to its surrounding detergent matrix and explains why the Fc segment was not co-extracted together with the F(ab′)_2_ domain.

Eight different extraction buffers comprising single amino acids, all at the same concentration (50 mM), pH (3.8) and salt concentration (125 mM NaCl) were studied. Amino acid buffers containing Val, Ile or Leu were thought to compete with the side chains of the Ab-fragments for hydrophobic interactions. Arg, Lys and Glu buffers were expected to compete for ionic and H-bond interactions and the His extraction buffer aimed at competing for metal chelation (Fig. 4E). The aforementioned 7 buffers were compared to Gly buffer, commonly used for IgG elution from Protein A columns ^34^ (Fig. 4E, lane 2). These competition experiments led to two conclusions: (i) Hydrophobic interactions exist between the captured F(ab′)_2_ fragment and the surrounding detergent matrix. This argument relies on the observation that extraction yields were improved when Gly buffer is replaced by either Val or Leu buffers (Fig. 4E, lanes 2,3 and 5). It is unclear to us why Ile buffer did not exhibit a similar pattern (Fig. 4E, lane 4). (ii) Positively charged side chains of the Fc fragment seem to be involved in binding to the aggregates since the presence of Arg, Lys or His buffers led to significant amounts of extracted Fc (Fig. 4E, lanes 6–8).

Additional evidence supporting conclusion (ii) was the finding that Fc fragment is not extracted with Glu buffer which carries a net negative charge at pH 3.8 (Fig. 4E, lane 9). Thus, Val and Leu buffers were found to be superior, with respect to purity and overall yield, to all other extraction buffers studied (Fig. 4E, lanes 3 and 5).

Process efficiency was further tested with Leu buffer, as the preferred extraction buffer, but in a more realistic environment containing impurities present in *E. coli* lysate (Fig. 4F). *E. coli* lysate was chosen as the default bacterial host cell protein background, since the identity of the actual expression cell culture was not provided to us. Both Tween-60 and Brij O-20 detergent aggregates efficiently excluded *E. coli* bacterial proteins from the F(ab′)_2_ fragments recovered and generated reasonably pure protein preparations (Fig. 4F, lanes 3–6). However, process yield decreased (~ 50–55%, by densitometry) due to the competition of bacterial proteins with the F(ab′)_2_ fragment for binding to the available surfaces of detergent aggregates. This argument was supported by the observation of a correlation between the amount of added bacterial impurities and process yield. The more impurities added the lower the overall yield (not shown). Quantitation of the amount of aggregates that dissociate during the extraction of the F(ab′)_2_ fragment was evaluated by comparing the intensity of the detergent aggregate band observed along with the extracted F(ab′)_2_ fragment to the band intensity of calibrated amounts of the same detergent aggregates. The observed degree of dissociation of Tween-60 and Brij-O20 micellar aggregates was found to be approx. 1wt% for both detergents (Figure S2). Thus, process efficiency would have to be determined on a case-by-case basis. It is also obvious that in order for the purification protocol presented here to be useful in a clinical setting, Strategy II would have to be followed by a chromatographic step (e.g. ion exchanger) which is capable of excluding very low levels of protein impurities as well as leached components deriving from the detergent scaffold per se, i.e. detergent monomers, chelators and Fe^2+^ ions.

The impact of this study on the field of Ab-fragment purification would increase if similar results could be demonstrated with other IgG's. We therefore evaluated the same purification protocol of Strategy II on polyclonal human IgG (hIgG). The IdeS cleavage pattern was very similar to that obtained with the mAb. Two major bands, due to F(ab′)_2_ and Fc were clearly observed, though the Fab band was highly diffuse (Supplementary, Figure S1-A, lane 3). The observed extraction efficiency using the same eight buffers was difficult to quantitate due to highly diffused bands representing a wide range of sequences present in a polyclonal antibody preparation (i.e. hIgG) but demonstrated again that best extraction buffers were: Val and Leu (Supplementary, Figure S1-B, lanes 3 and 5) while positively charged amino-acids buffers coextracted the Fc domain (Supplementary, Figure S1-B, lanes 6–8) consistent with our findings with the mAb (Fig. 4E, lanes 6–8). Quantitation of process yields, indicated that extraction buffers comprising single hydrophobic amino acids or Gly (as an exception), are most efficient, resulting in moderate to good recovery (70–87%) (Table 1).

**Table 1:** Overall recovery yields of F(ab′)_2_ via Strategy II from either a mAb or polyclonal human IgG (hIgG).

	Gly	Val	lle	Leu	Arg	Lys	His	Glu
mAb*	72–84	70–87	73–82	76–86	59–76	60–71	65–76	61–72
hlgG*	52–63	50–64	32–55	53–68	33–61	31–60	34–49	53–68

*Values represent at least 3 independent experiments.

**Figure 5:**
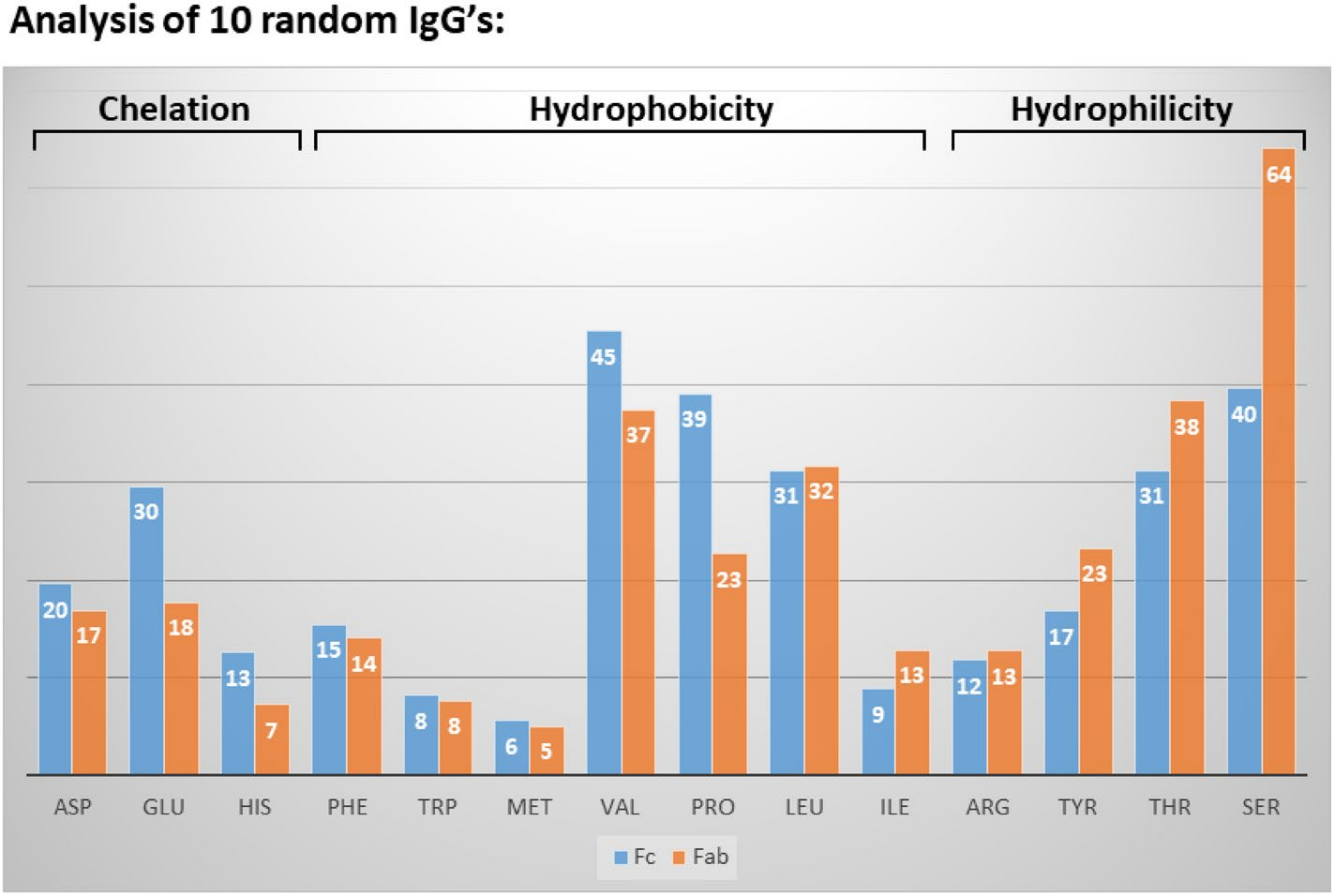
Comparison of amino acid compositions of Fc and Fab domains for 10 randomly chosen IgG’s. Number of amino acids present in either the Fc or Fab domains capable of binding metals (Chelation, LHS); characterized as hydrophobic (center) or hydrophilic (RHS), is indicated. Sequences were retrieved from the UniProt database (https://www.uniprot.org/). Values for Trp were rounded off, explaining the differences in bar heights. Additional information regarding these antibody sequences can be found in the Supplementary section (Table 1).

A plausible explanation for the greater tendency of the Fc fragment to bind to amphiphilic detergent aggregates in comparison to the F(ab′)_2_ domain relies on amino acid analysis (Fig. 5). We compared the amino acid composition of the Fc and the F(ab′)_2_ fragments of 10 randomly chosen IgG’s (exhibiting diverse specificities) and found that the Fc domain is richer in residues capable of participating in metal chelation (i.e. Glu, His) and has a larger number of the hydrophobic amino acids, Val and Pro, while the Fab domain is richer in Thr and Ser (Fig. 5).These findings suggest that the Fc domain is more likely to form chelation interactions with the [(batho)_3_:Fe^2+^] amphiphilic complex via an excess of imidazole (His) and carboxylate (Glu) side chains, in parallel with a larger number of van der Waals (induced dipole–dipole) interactions due to the Val and Pro residues. At the same time, the Fab arms may be more polar and hydrophilic (due to the abundance of Ser + Thr) explaining their lower binding affinity to hydrophobic surfaces present in the detergent aggregates.

Three previous studies^27–29^ demonstrated that efficient antibody purification is achieved only when non-ionic detergent micelles form a new and larger oil-rich phase upon micellar conjugation with the [(bathophenanthroline)_3_:Fe^2+^)] complex. This suggests that van der Waals and/or entropically driven hydrophobic interactions are at the heart of the IgG binding mode in our purification strategy. Tween, Brij, and Triton families are the most successful, while Pluronic F-127, which lacks a hydrophobic anchor, is less so. And there are size limitations as well, in that the hydrodynamic size of the micellar aggregate must be ≥ 1 micron. It seems reasonable to argue that, similar interactions should play a controlling role in binding the F(ab′)_2_ domain to the aggregate.

**Figure 6:**
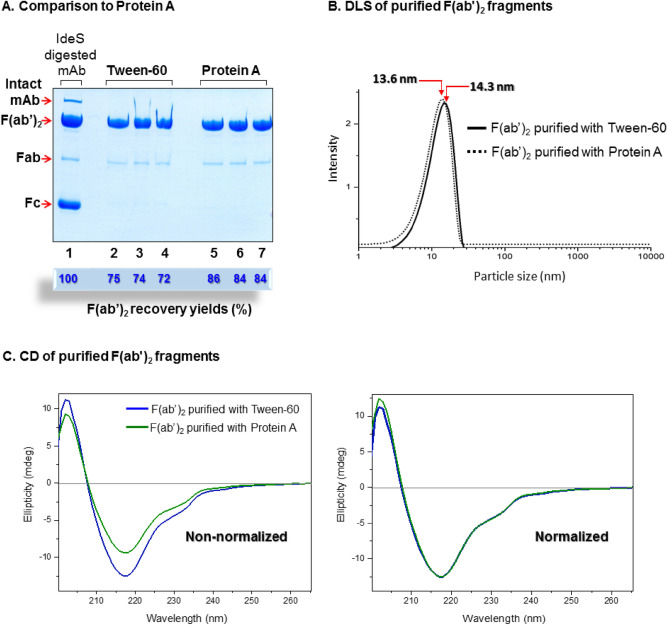
Comparison of detergent aggregate purification and Protein A purification of fragments from IdeS digested mAb. (**A**) Lane 1: IdeS digested mAb; lanes 2–4: F(ab′)_2_ recovered after incubation of IdeS digested mAb with Tween-60 aggregates and following extraction as described in the Experimental section; lanes 5–7: flow-through composition after IdeS digestion (as shown in lane 1) and followed by removal of the F_c_ domain on a Protein A column. Overall F(ab′)_2_ recovery yields are indicated below each lane. The gel is Coomassie stained. *E. Coli* lysate was not added. (**B**) Dynamic light scattering (DLS) of F(ab′)_2_ fragments purified with either Tween-60 aggregates or a Protein A spin column. (**C**) Circular dichroism (CD) spectra of F(ab′)_2_ fragments purified with either Tween-60 aggregates or a Protein A spin column (Both the non-normalized and normalized spectra are presented. Normalization of the spectra is performed at 218 nm).

Comparison with Protein A chromatography, in the absence of *E. coli* lysate background, allowed comparisons of purity and yield and determining whether recovered F(ab′)_2_ fragments had preserved their secondary structure during interaction with the detergent aggregates. Therefore, the same IdeS cleaved mAb preparation used throughout this study was subjected to either purification with Tween-60 aggregates or to a Protein A column responsible for binding the cleaved Fc domains, thereby excluding these from supernatant containing the cleaved F(ab′)_2_ fragments. The results indicate that both methods were efficient in removing the Fc fragment from the initial IdeS cleaved antibody preparation and F(ab′)_2_ fragments with essentially identical purity were observed (Fig. 6A, lanes 2–4 vs. lanes 5–7). Small amounts of Fab domain remaining in the Protein A samples (Fig. 6A, lanes 5–7) are likely generated when running the gel under reducing conditions. The presence of β-mercaptoethanol may reduce S–S bonds in the F(ab)_2_ domain and generate the observed Fab segment band, rather than its being due to the purification protocol itself; this comparison was performed multiple times and led to identical results.

However, recovery yields obtained using the Protein A column were higher (84–86% vs. 72–75%) due to the minimal interaction of the F(ab′)_2_ fragment with the Protein A resin in comparison to its stronger binding affinity to Tween-60 aggregates. Dynamic light scattering (DLS) analysis of purified F(ab′)_2_ fragments show that both routes generated particles with very similar hydrodynamic size: 13.6 nm for Tween-60 and 14.3 nm for Protein A (Fig. 6B). Importantly, no evidence for protein aggregation was observed up to 10,000 nm (Fig. 6B).

As we have noted in the Experimental section, this study uses a commercial (but proprietary) mAb, for which the amino acid sequence and medicinal use were not provided. Consequently, analytical assays commonly required in the antibody purification field—enzyme-linked immunosorbent assay (ELISA) analysis as well as calculation of the accessible surface area (ASA) of the purified F(ab′)_2_ fragments, could not be performed. Nevertheless, far-UV circular dichroism (CD) spectroscopy, a very sensitive tool for determination of secondary structure and folding properties of proteins, was available as an excellent alternative. CD spectroscopy has been widely used to determine the folding state of an isolated native protein, expressed purified protein or mutated protein^35^. As such, CD spectroscopy was found to display marked sensitivity toward the detection of conformational changes which could modulate the stability of therapeutic antibodies during process development, as well as product characterization^36^. Indeed, the structural information provided by CD was able to detect the differences in antigen binding specificity which were conferred by the Ab constant heavy domain^36^. Thermal scanning CD has been used as a high throughput screening tool for examining therapeutic mAb structural stability in various formulation and downstream buffers. By monitoring mAb aggregational states induced in high salt or low pH, aggregation mechanisms of mAb have been elucidated by CD^37^. Finally, far UV-CD spectroscopy has been shown to successfully correlate mAb secondary structure with ligand binding at low pH^38^. The circular dichroism spectra of both of our F(ab′)_2_ samples could be superimposed on each other (Fig. 6C) and provided evidence for the preservation of the commonly observed antibody secondary structure: anti-parallel beta pleated sheet and its characteristic dominant negative peak at ~ 218 nm^39,40^.

## In conclusion

Aggregates consisting of non-ionic detergent (Tween-60 or Brij O-20) micelles, the amphiphilic chelator bathophenanthroline and Fe^2+^ ions, bind the Fc domain of IgG’s with greater affinity than the F(ab′)_2_ segment, thereby permitting fragment separation. Pure F(ab′)_2_ preparations (≥ 90%) are obtained without chromatography or specific ligands in the absence or, more importantly, in the presence of *E. coli* lysate. As noted above, this study uses a commercial mAb, for which the amino acid sequence and medicinal use were not provided. As a result, analytical assays commonly required in the antibody purification field—enzyme-linked immunosorbent assay (ELISA) analysis as well as calculation of the accessible surface area (ASA) of the purified F(ab′)_2_ fragments, could not be performed. However, despite the absence of these important data that would, if present, have confirmed preservation of antigenic binding specificity and mode of interaction with the detergent matrix, we believe that presenting this novel purification platform for F(ab′)_2_ fragments justifies sharing the current data. The practical utility of our platform for other Ab-fragments (e.g. Fab, scFv and single domain antibodies) remains to be demonstrated.

## Supplementary Information

Supplementary Information.
