# Supplementary material for: Purification of antibody fragments via interaction with detergent micellar aggregates
Source: Sci Rep. 2021 Jun 3;11:11697. doi: 10.1038/s41598-021-90966-1 (PMC8175343; doi:10.1038/s41598-021-90966-1)
Supplement: Supplementary file 1 — Supplementary Information. [file 41598_2021_90966_MOESM1_ESM.docx]

**Purification of** **antibody fragments *via* interaction with detergent micellar aggregates**

**Supplementary**

Gunasekaran Dhandapani,^1^ Ellen Wachtel,^2^ Ishita Das,^2^ Mordechai Sheves ^2^ and Guy Patchornik^1*^

^1^ Department of Chemical Sciences, Ariel University, 70400, Ariel, Israel.

^2^ Faculty of Chemistry, Weizmann Institute of Science, 76100, Rehovot, Israel.

*Corresponding author:

Email: [guyp@ariel.ac.il](mailto:guyp@ariel.ac.il)

**
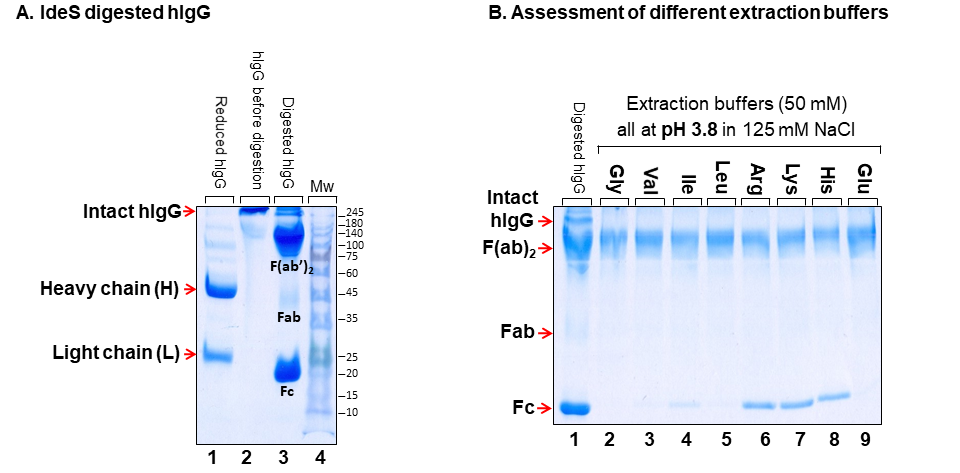
**

**Figure S1: Implementation of “Strategy II” on human IgG (hIgG).** **A.** Lane 1: reduced heavy and light chains of hIgG; lane 2: intact hIgG not subjected to enzymatic cleavage by IdeS; lane 3: as in lane 2, but following IdeS digestion; lane 4: molecular weight markers. **B.** Lane 1: IdeS digested hIgG mixture; lanes 2-9: F(ab')_2_ recovered from Tween-60 aggregates using the extraction conditions indicated. Gels are Coomassie stained. *E. coli* lysate was not added.


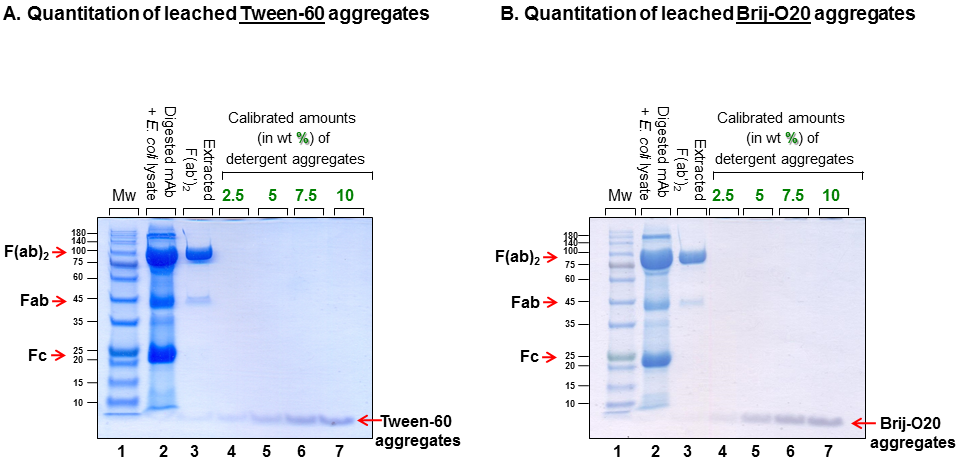


**Figure S2: Assessment of dissociated aggregates during extraction of F(ab’)_2_ at pH 3.8. A.** Lane 1: Molecular weight markers; lane 2: Mixture of the mAb , subjected to enzymatic cleavage by IdeS, and added *E. coli* lysate; lane 3: F(ab')_2_ domain recovered from Tween-60 aggregates using extraction conditions described in the Experimental section; lanes 4-7: calibration markers for known amounts of Tween-60 aggregates devoid of any protein. **B.** As in **A** but in the presence of Brij-O20 aggregates. Fab and Fc stand for antigen binding fragment and crystallizable fragment, respectively. Gels are Coomassie stained.

**Analyzed antibody sequences with specified domains**

| **Fab Heavy chain** | | | | |
| --- | --- | --- | --- | --- |
| S.No | Entry | Name | Length | Species |
| 1 | 4UAO_C | Chain C, Immunoglobulin R31c2 Vh And Ch1 Regions | 223 aa | *Rattus norvegicus* |
| 2 | 6H5N_C | Chain C, Antibody 85RF45.1 heavy chain | 220 aa | *Rattus norvegicus* |
| 3 | 6MNQ_H | Chain H, Ab DH727.2 heavy chain Fab fragment | 221 aa | *Macaca mulatta* |
| 4 | 6P8N_B | Chain B, P-p1f1 Heavy Chain | 226 aa | *Mus musculus* |
| 5 | 4Q2Z_A | Chain A, Heavy Chain Of Fab Fragment Of Hiv Vaccine-elicited Cd4bs- Directed Antibody | 233 aa | *Macaca mulatta* |
| 6 | 6P8M_H | Chain H, P-p3b3 Heavy Chain | 233 aa | *Mus musculus* |
| 7 | 4KQ3_H | Chain H, CNTO3186 heavy chain | 226 aa | *Homo sapiens* |
| 8 | 6C6Z_C | Chain C, Antibody CDC2-C2 heavy chain | 239 aa | *Homo sapiens* |
| 9 | 4NWT_H | Chain H, Ape1531 Ab Fab Heavy Chain | 233 aa | *Homo sapiens* |
| 10 | 6AL0_H | Chain H, Heavy chain of antigen binding fragment, Fab of NZ-1 | 219 aa | *Rattus norvegicus* |
| **Fab light chain** | | | | |
| S.No | Entry | Name | Length | Species |
| 1 | 5HBT_C | Chain C, Fab35, Light Chain | 213 aa | *Rattus norvegicus* |
| 2 | 1ZAN_L | Chain L, Crystal Structure Of Anti-Ngf Ad11 Fab | 214 aa | *Rattus norvegicus* |
| 3 | 4N9G_B | Chain B, Antibody 17HD9, Light Chain | 215 aa | *Macaca mulatta* |
| 4 | 6TYB_L | Chain L, NEUTRALIZING ANTIBODY ITS90.03 FAB LIGHT CHAIN | 214 aa | *Macaca mulatta* |
| 5 | 6P8N_D | Chain D, P-p1f1 Light Chain | 216 aa | *Macaca mulatta* |
| 6 | 6P8M_L | Chain L, P-p3b3 Light Chain | 216 aa | *Mus musculus* |
| 7 | 4JZJ_B | Chain B, Fab Light Chain | 220 aa | *Mus musculus* |
| 8 | 5IJK_C | Chain C, 1E03 Fab fragment light chain | 221 aa | *Homo sapiens* |
| 9 | 6B5L_L | Chain L, CIS43 Fab Light Chain | 220 aa | *Homo sapiens* |
| 10 | 4NWT_L | Chain L, Ape1531 Ab Fab Light Chain | 220 aa | *Homo sapiens* |
| **Fc Chain** | | | | |
| S.No | Entry | Name | Length | Organism |
| 1 | 3V7M_A | Chain A, Ig Gamma-1 Chain C Region | 209 aa | *Homo sapiens* |
| 2 | 5U4Y_A | Chain A, IgG1 fc | 212 aa | *Homo sapiens* |
| 3 | 1OQO_A | Chain A, Immunoglobulin Gamma-1 Heavy Chain Constant Region | 212 aa | *Homo sapiens* |
| 4 | 4QGT_A | Chain A, Hepatitis B Virus Receptor Binding Protein | 233 aa | *Homo sapiens* |
| 5 | 1FC1_A | Chain A, Fc Fragment | 224 aa | *Homo sapiens* |
| 6 | 5DJ0_A | Chain A, Ig Gamma-1 Chain C Region | 227 aa | *Homo sapiens* |
| 7 | 3AGV_A | Chain A, Crystal Structure of A Human Igg-Aptamer Complex | 211 aa | *Homo sapiens* |
| 8 | 5U52_A | Chain A, IGG1 FC | 209 aa | *Homo sapiens* |
| 9 | 3HKF_A | Chain A, Murine Un-glycosylated Igg Fc Fragment | 214 aa | *Mus musculus* |
| 10 | 6D4I_A | Chain A, Fc fragment of IgG2 | 223 aa | *Macaca mulatta* |

**Table 1:** Identification of the antibody molecules included in the <https://www.uniprot.org/> database, from which the amino acid sequences of the Fc and F(ab')_2_ domains presented in Figure 5, were retrieved.
